# Facteurs associés à l’acceptation de la vaccination contre la Covid-19 dans la zone de santé Kokolo à Kinshasa, République démocratique du Congo (RDC)

**DOI:** 10.48327/mtsi.v5i4.2025.704

**Published:** 2025-12-22

**Authors:** Levis AMISI KENGEA, Winnie MASAMBA BIKOKI, Jean-Claude NSINGA BUNGIENA, Jean-Jacques KAPE KALUME

**Affiliations:** 1 Zone de santé Kokolo, Corps de santé militaire, Forces armées de la RDC, Kinshasa, RDC; 2 Hôpital militaire central, Corps de santé militaire, Forces armées de la RDC, Kinshasa, RDC; 3 Corps de santé militaire, Forces armées de la RDC, Kinshasa, RDC

**Keywords:** Communauté, Vaccination Covid-19, Zone de Santé Kokolo, Kinshasa, République démocratique du Congo, Afrique subsaharienne, Community, COVID-19 vaccination, Kokolo Health Zone, Kinshasa, Democratic Republic of Congo, Sub-Saharan Africa

## Abstract

**Contexte:**

La vaccination est l’une des stratégies préventives majeures dans la conduite de la riposte contre la Covid-19. Cependant, l’adhésion à la vaccination demeure un défi à relever dans les communautés notamment celle de la zone de santé (ZS) de Kokolo (Kinshasa, RDC) où la couverture vaccinale reste en dessous de 40%. Doù l’intérêt d’identifier les facteurs d’acceptation de la vaccination contre la Covid-19 chez les chefs de ménage ou leurs conjoints.

**Méthodologie:**

Une étude transversale à visée analytique a été réalisée de janvier 2021 à décembre 2024 auprès des 399 chefs de ménage (ou conjoints), sélectionnés par échantillonnage probabiliste à trois degrés. Les données ont été collectées par interview sur un questionnaire structuré sur *KoboCollect* et analysées sur Epi info 7.2.6.0.

**Résultats:**

Dans l’ensemble, la population de la ZS a déclaré avoir déjà entendu parler de la Covid-19 ainsi que de la vaccination anti-Covid-19. Parmi les personnes interrogées, 79,9% (soit 319 sur 399) considéraient que la vaccination était efficace. Le fait de n’avoir jamais vu un mort de Covid-19, ni aucun cas déclaré dans la ZS étaient les raisons principales de la non-acceptation de la vaccination. En analyse multivariée, l’acceptation de la vaccination était associée au fait d’être militaire, d’avoir des connaissances sur cette maladie et sur la vaccination existante.

**Conclusion:**

Les connaissances sur la Covid-19 et la vaccination anti Covid-19, l’implication des autorités politico-administratives, des autorités militaires et des leaders communautaires, la sensibilisation et l’organisation de sites de vaccination rapprochés doivent être pris en compte dans l’élaboration des stratégies pour favoriser l’acceptation de la vaccination. L’utilisation des médias est la principale voie d’information et de sensibilisation dans cette ZS.

## Abstract (en)

**Context:**

Vaccination is a key preventive strategy in the response to the SARS-CoV-2 pandemic. However, ensuring vaccination adherence remains challenging, particularly in the Kokolo health zone (HZ) in Kinshasa, Democratic Republic of the Congo (DRC), where vaccination coverage remains below 40%. This study aims to identify the factors influencing acceptance of the vaccine among heads of households or their spouses.

**Methodology:**

From January 2021 to December 2024, a cross-sectional analytical study was conducted among 399 heads of households (or spouses) selected by three-stage probability sampling. Data were collected through interviews using a structured questionnaire on the *KoboCollect* application and analyzed using Epi Info 7.2.6.0.

**Results:**

Overall, the HZ population reported having heard of both the COVID-19 and its vaccination. Among those surveyed, 79.9% (319 out of 399) considered the vaccine to be effective. The main reasons for not accepting vaccination were never having seen a death or reported case of the disease in the HZ. In a multivariate analysis, acceptance of the vaccine was associated with military service and knowledge of the disease and existing vaccines.

**Conclusion:**

When developing strategies to promote Covid-19 vaccination acceptance, knowledge about the disease and existing vaccination, the involvement of political and administrative authorities, military authorities, and community leaders, awareness-raising, and the organization of nearby vaccination sites must be taken into account. Media is the main channel for information and awareness-raising in this HZ.

## Introduction

La Covid-19 a été un défi majeur de santé publique en République démocratique du Congo (RDC), en particulier à Kinshasa et dans la zone de santé (ZS) Kokolo [1-8,10-13,16-20,22-24]. Depuis avril 2021, le pays a recensé 92 632 cas confirmés et 1 357 décès, soit une létalité de 1,47% [19]. Malgré l’instauration de la vaccination comme stratégie principale, l’adhésion est restée faible: 6,4% des habitants de Kinshasa ont reçu une première dose et 4,2% ont été complètement vaccinés. Dans la ZS Kokolo, les 27 aires de santé ont été touchées, avec un total de 3 829 cas et 28 décès. Le manque d’adhésion vaccinale, malgré la vaccination de routine, a limité l’efficacité de la riposte. Contrairement à d’autres pays comme les Seychelles ou l’Île Maurice, la RDC n’a pas atteint les seuils de couverture souhaités [4,8, 12,21,24]. Cette étude vise à identifier les facteurs favorisant l’acceptation de la vaccination afin d’adapter la communication et d’améliorer la couverture vaccinale. Les objectifs spécifiques étaient de décrire les caractéristiques sociodémographiques et économiques des chefs de ménage ou de leurs conjoints, de déterminer leur niveau de connaissances, leurs attitudes et leurs pratiques en rapport avec la Covid-19 et la vaccination anti-Covid-19, et d’identifier les facteurs associés à l’acceptation de la vaccination.

## Méthodes

Il s’agit d’une étude transversale à visée analytique réalisée dans la ZS Kokolo de janvier 2021 à fin décembre 2024. Cette zone, urbano-rurale et militaire, est l’une des 35 ZS de la Division provinciale de la santé (DPS) de Kinshasa. Elle comprend 27 aires de santé (AS) et couvre une population de 653 645 habitants, avec l’hôpital militaire central comme hôpital général de référence.

Celui-ci disposait d’un centre de traitement Covid-19 (CTCo) pour les cas déclarés dans le district de Funa, l’un des quatre districts de la ville de Kinshasa. Sur décision des autorités sanitaires nationales comme de la province, la ZS Kokolo ne disposait pas de vaccinodrome pour vacciner 7 jours sur 7 et 24h sur 24 contre la Covid-19. Cependant, il y avait 27 sites de vaccination à raison d’un site par AS, au sein du centre de santé. Ces derniers ne fonctionnaient que de 8h à 16h, avec mission de vacciner une cinquantaine de personnes par jour.

La population d’étude était des chefs de ménage ou leurs conjoints, qui ont constitué les unités statistiques.

Les critères d’inclusion étaient d’avoir 18 ans ou plus et de vivre dans le ménage depuis plus de 6 mois.

Les critères de non-inclusion étaient les adultes dont l’état mental ne permettait pas de fournir des réponses et l’absence de consentement à la participation de l’étude. Il n’y avait pas de critère d’exclusion.

La taille minimale de l’échantillon était de 399 personnes avec une méthode d’échantillonnage probabiliste à 3 degrés. Au premier degré, les AS ont été choisies par échantillonnage aléatoire simple (en utilisant la liste des AS de la ZS comme base de sondage). Au deuxième degré, les cellules d’animation communautaire (CAC) ont été sélectionnées dans la liste des CAC de chaque AS retenue. Au troisième degré, dans chaque CAC, les ménages ont été sélectionnés selon le pas de sondage et le chef de ménage ou son conjoint a été interviewé.

La collecte des données a été faite par entretien direct avec un questionnaire préalablement testé dans la ZS de Bandal. Celle-ci est voisine du camp Kokolo avec lequel elle partage un fort mouvement de la population dont quelques militaires et civils qui y résident.

Le traitement et l’analyse des données ont été effectués en respectant la confidentialité et l’anonymat des sujets interviewés ainsi que le contrôle qualité des données enregistrées sur Excel version 2016. L’analyse a été effectuée avec le logiciel Epiinfo 7. 2. 6. O.

Les résultats ont été présentés sous forme de tableaux, les données quantitatives sous forme de moyenne avec son écart type, ou de médiane avec son étendue et les données catégorielles sous forme de proportion.

Toutes les autorisations auprès des autorités militaires et sanitaires de la ZS Kokolo et des structures sanitaires ont été obtenues.

La participation à l’enquête a été volontaire et sans contrainte, laissant à chaque répondant le loisir de participer ou pas à l’étude et de répondre librement aux questions. Un consentement éclairé écrit a été obtenu auprès des enquêtés. La confidentialité des informations collectées a été garantie. Les enquêtés avaient la possibilité de se retirer de l’étude s’ils le désiraient.

## Résultats

Au total, 399 personnes ont été interviewées. Plus de la moitié étaient des femmes. Les plus de 54 ans étaient en faible proportion. Près de 7 personnes sur 10 avaient un niveau d’éducation secondaire, plus de 6 sur 10 étaient des militaires. Il n’y avait pas de différence statistique entre les répondants ayant 6 enfants ou moins, et ceux ayant plus de 6 enfants dans le ménage. Plus de 6 personnes sur 10 venaient à pied au site de vaccination (Tableau I). Dans l’ensemble, la quasi-totalité des personnes interrogés a déclaré avoir déjà entendu parler de Covid-19 (Tableau II) et de la vaccination contre la Covid-19, la source principale d’information étant la télévision. Sept sur dix connaissaient l’importance de la vaccination ainsi que son efficacité. Une bonne partie (90% des répondants) avait des connaissances sur les moyens de lutte. La plupart connaissait les différents effets indésirables de la vaccination (Tableau III). Près de neuf sur dix avaient de bonnes connaissances sur la Covid-19 et sur la vaccination (Tableau IV).

**Tableau I:** Caractéristiques sociodémographiques des répondants Table I: Sociodemographic characteristics of respondents

Variables	Effectif (n=399)	Proportion (%)
Genre		
féminin	201	50,4
masculin	198	49,6
Âge (années)		
18-29 ans	117	29,3
30-41 ans	153	38,3
42-53 ans	88	22,1
≥ 54 ans	41	10,3
État marital		
marié(e)	188	47,1
célibataire	152	38,1
veuf(ve)	37	9,3
divorcé(e)	22	5,5
Niveau d’études		
primaire	214	53,6
secondaire	132	33,1
supérieur	47	11,8
autres (à préciser)	6	1,5
Travail avant la Covid-19		
militaire	261	65,4
dépendant militaire	59	14,8
activité libérale	49	12,3
pas d’occupation	29	7,3
autres (personnel civil)	1	0,3
Travail actuel		
militaire	263	65,9
dépendant militaire	64	16,1
activité libérale	45	11,3
pas d’occupation	24	6,0
autres (personnel civil)	3	0,7
Nombre d’enfants dans le ménage		
≤ 6 enfants	201	50,4
> 6 enfants	198	49,6
Moyen de transport		
marche	322	80,7
transports en commun	62	15,5
transport personnel	4	1,0

**Tableau II:** Répartition des répondants selon leurs connaissances sur la Covid-19

Variables	Effectif (n=399)	Proportion (%)
Connaissance de l’existence de la Covid-19		
oui	399	100
non	0	0
Sources d’information		
télévision	212	53,1
radio	94	23,6
réseaux sociaux	72	18,0
entourage	54	13,5
relais communautaire	99	24,8
prestataire de soins	31	7,8
Moyens de transmission du virus		
contact direct avec une personne malade	362	90,7
transmission aéroportée	308	77,2
contact avec les gouttelettes de salive infectée	227	56,9
contact avec les objets souillés	64	16,0
Symptômes de la Covid-19		
fièvre	381	95,5
toux	368	92,2
essoufflement	367	91,9
maux de tête	363	90,9
douleurs musculaires	336	84,2
fatigue	312	78,2

**Tableau III:** Répartition des répondants selon leurs connaissances sur la vaccination contre la Covid-19

variables	Effectif (n=399)	Proportion (%)
Connaissance de l’existence de la vaccination contre la Covid-19		
oui	397	99,5
non	2	0,5
Sources d’information		
télévision	212	53,1
radio	99	24,8
réseaux sociaux	91	22,8
entourage	82	20,5
relais communautaire	31	7,8
prestataire de soins	54	13,5
Importance de la vaccination		
connaît	286	71,7
ne connaît pas	113	28,3
Efficacité du vaccin		
fortement d’accord	197	49,4
d’accord	122	30,6
pas du tout d’accord	49	12,3
ne sait pas	31	7,8
Moyens de lutte		
masque facial	374	93,7
lavage régulier des mains	371	92,9
éviter de saluer à la main	360	90,2
éviter le contact physique	348	87,2
garder au moins une distance d’un mètre	339	84,9
utiliser le coude	336	84,2
prier	317	79,4
utiliser les plantes	258	64,7
aller dans une structure de soins dès les premiers symptômes	254	63,7
être complètement vacciné	205	51,3
Effets indésirables de la vaccination		
maux de tête	327	81,9
fièvre	324	81,2
douleurs/courbatures	305	76,4
diarrhée	264	66,2
nausées/vomissements	159	39,8
asthénie physique	105	26,3

**Tableau IV:** Répartition des répondants selon leurs attitudes et pratiques face à la Covid-19

Variables	Effectif (n=399)	Proportion (%)
Croire à l’existence du Covid-19	343	85,9
Raisons de non-croyance		
n’a jamais vu de morts de la Covid-19	58	14,5
n’a jamais vu un cas de Covid-19 dans la ZS	51	12,8
n’a jamais vu des personnes guéries de cette maladie	47	11,8
Prédisposition des enquêtés à contracter l’infection de Covid-19 lorsqu’ils sont exposé à l’agent pathogène	266	66,7
Raisons de non-prédisposition à contracter l’infection de Covid-19		
pas de contact avec les gens infectés	78	19,5
n’être pas être riche, c’est la maladie des riches	63	15,8
la Covid-19 n’existe pas	56	14,0
non-résidence dans les AS touchées	39	9,8
Acceptation du vaccin	319	79,9
Personnes complètement vaccinées	184	46,1
Motivations pour la vaccination		
convictions personnelles	83	20,8
par la communauté	37	9,3
par les professionnels de santé	28	7,0
par les membres de famille	19	4,8
par les autorités militaires et civiles	17	4,3
Éléments influençant la vaccination		
implication de la communauté	232	58,1
exemple donné par les autorités et des leaders de la communauté	229	57,4
lieu d’habitation	229	57,4
rejet de la peur	224	56,1
communication reçue des autorités et des leaders de la communauté	223	55,9
niveau socioéconomique	221	55,4
niveau de scolarisation	219	54,9

Plus de huit personnes sur dix considéraient que la vaccination était efficace. Le fait de n’avoir jamais vu de personne décédée de Covid-19 ni de cas de contamination par le virus étaient les raisons principales de la non-acceptation de se faire vacciner. La proportion de vaccinés était de 25,1% et le taux d’acceptation au sein de la communauté était de 9%. Ne pas avoir peur de se faire vacciner avec un vaccin nouvellement mis sur le marché, l’exemple donné par les autorités et des leaders de la communauté et la communication reçue des autorités et des leaders de la communauté étaient les éléments influençant les plus cités pour l’acceptation du vaccin.

Plus de la moitié des répondants avait déjà vu une personne se faire vacciner contre la Covid-19 et plus de trois sur dix de ces personnes étaient des membres de leur famille. Dans l’ensemble, la quasi-totalité des répondants n’avait jamais vu une personne décédée de Covid-19.

Parmi les différentes mesures préconisées pour éviter la Covid-19, le lavage régulier des mains était la mesure la plus citée. Par contre, seulement le quart des répondants pensait que la vaccination serait une bonne mesure. La peur de complications était la raison citée pour justifier la non-adhésion au vaccin. Près de quatre personnes sur dix ont cité la sensibilisation comme moyen pour améliorer l’acceptation du vaccin dans la communauté (Tableau V).

**Tableau V:** Tableau V: Répartition des répondants selon leurs pratiques face à la vaccination

Variables	Effectif (n=399)	Proportion (%)
Mesures prises pour ne pas avoir la Covid-19		
se laver régulièrement les mains	339	84,9
porter un masque	294	73,7
éviter la salutation à la main	272	68,2
éviter de tousser	261	65,4
garder une distance d’au moins 1 à 2 m	207	51,9
se faire vacciner	184	46,1
éviter les endroits publics	167	41,8
réduire la fréquentation des personnes non vaccinées	135	33,8
Difficultés rencontrées vis-à-vis de la vaccination		
la peur de complications liées au vaccin	158	39,6
les fausses informations répandues autour de la vaccination	153	38,3
les croyances, us et coutumes	137	34,3
une sensibilisation discontinue de la population	132	33,1
une bonne communication des APA* et des leaders de la communauté	76	19,0
un bas niveau socioéconomique	52	13,0
un long trajet pour arriver à la structure de soins	44	11,0
le stock insuffisant des vaccins	29	7,3
Propositions pour améliorer l’acceptabilité		
sensibilisation ciblée et durable visant le changement des comportements, le renforcement des connaissances et la mobilisation de tous autour de la riposte contre Covid-19	159	39,9
fabrication de nos propres vaccins	84	21,1
développement des intrants (médicaments et équipements de protection)	38	9,5
augmentation du nombre de sites de vaccination	19	4,8

* APA: autorités politico-administratives

En analyse bivariée, six variables sont en relation avec l’acceptation du vaccin: le statut de militaire, les connaissances sur la Covid-19, celles sur la vaccination, l’implication des autorités politicoadministratives (APA) et des leaders communautaires, la sensibilisation sur la vaccination contre la Covid-19, la proximité et l’accessibilité du site de vaccination (Tableau VI).

**Tableau VI:** Relation entre facteurs et acceptation du vaccin

Variables	Accepte le vaccin		OR	IC 95%	p
	Oui (n=319)	Non (n=80)			
Genre du répondant					
féminin	160	41	1	(0,743-2,007)	0,431
masculin	159	39	1,221		
Âge					
<54 ans	308	50	1	(0,046-2,823)	0,330
≥55 ans	II	30	0,359		
Profession					
pas d’occupation	18	6	1		
dépendant militaire	58	6	4,500	(0,601-33,708)	0,143
militaire	207	56	18,50	(2,457-36,320)	0,005
activité libérale	35	10	2,100	(0,817-34,615)	0,268
autres (personnel civil)	1	2	6,250	(1,016-38,448)	0,051
Connaissances sur la Covid-19					
connaît	307	51	0,196	(0,099-0387)	< 0,001
ne connaît pas	12	29	1		
Connaissances sur la vaccination					
connaît	302	61	4,036	(3,052-5,312)	0,003
ne connaît pas	17	19	1		
Implication des APA, des autorités militaires et des leaders communautaires					
oui	262	51	1	(0,224-0,675)	< 0,001
non	57	29	0,393		
Sensibilisation					
oui	299	62	4,036	(2,867-6,254)	< 0,001
non	20	18	1		
Proximité et accessibilité du site					
non éloigné	291	39	1	(0,265-0,731)	<0,001
éloigné	28	41	0,431		

* APA: autorités politico-administratives/ PPAs: Political and administrative authorities

La cote de la profession (être militaire) est 18,5 fois plus élevée comparativement aux chômeurs. La cote était 5 fois plus élevée pour les personnes ayant des connaissances sur la Covid-19, par rapport à celles qui n’en avaient pas. La cote était 4 fois plus élevée pour les personnes ayant des connaissances sur la vaccination, par rapport à celles qui n’en avaient pas. La non-implication des APA, des autorités militaires ou des leaders communautaires réduisait de 2,6 la probabilité de se faire vacciner. La sensibilisation multipliait par 4 la cote de se faire vacciner par rapport à la non-sensibilisation.

Avec la régression logistique, trois variables étaient significativement associées à l’acceptation du vaccin: être militaire, avoir des connaissances sur la Covid-19 et sur la vaccination (Tableau VII).

**Tableau VII:** Répartition des répondants selon les facteurs associés à l’acceptation du vaccin

Caractéristiques	ORa (95%)	p (LR test)
Être militaire	3,09 (1,03-5,15)	0,005*
Connaissances sur la Covid-19	2,2 (1,33-3,26)	< 0,001*
Implication des APA, autorités militaires et leaders communautaires	9,11 (0,96-87,95)	0,056
Connaissances sur la vaccination	2,67 (1,05-4,65)	0,029*
Sensibilisation	5,96 (0,88-11,01)	0,105
Proximité et accessibilité du site	7,04 (0,91-83,74)	0,057

Valeur Akaike information criterion: 500,954; Ora: odds ratio ajusté; LR: rapport de vraisemblance; * signification

## Discussion

La population d’étude connaissait largement la Covid-19 et sa vaccination, avec la télévision comme principale source d’information (53%). Plus de 80% des interrogés jugeait la vaccination efficace. Toutefois, ne jamais avoir vu de cas de Covid-19 ou de décès liés à ce virus dans la ZS expliquait en grande partie la réticence à se faire vacciner. L’acceptation vaccinale était 3 fois plus élevée chez les militaires. Elle était également plus élevée chez les personnes informées sur la maladie (2,2 fois) et sur la vaccination (2,6 fois). Mbongopasi *et al.* avaient trouvé que 51% de personnes de Kinshasa se considéraient informées surtout par la télévision [12]. Cependant, ces auteurs avancent un risque de désinformation suite à la propagation d’un mélange d’informations exactes et inexactes sur le sujet. Dans une étude similaire en Ouganda, les principales sources d’information étaient les organisations internationales de la santé comme le CDC et l’OMS [14-16].

La couverture vaccinale dans la ZS Kokolo atteint 46%, surtout chez les individus de moins de 54 ans résidant près des centres de santé où se trouvaient les sites de vaccination. Ce taux dépasse la moyenne nationale congolaise (8,3%) mais reste inférieur aux attentes. Plusieurs obstacles expliquent ce taux dans la ZS: absence de vaccinodrome, peu de campagnes de masse, logistique limitée et déplacements militaires fréquents. Les motifs de refus vaccinal incluent l’absence perçue de cas graves dans la communauté et une méfiance envers l’efficacité des vaccins, comme observé également dans la ZS de N’djili, l’une des communes de Kinshasa [2,3, 7,17,19,24].

Le scepticisme, alimenté par les rumeurs et le manque de confiance dans le système de santé, freine le passage de l’intention à l’acte. Pour y remédier, des messages clairs sur la sécurité et l’efficacité des vaccins sont indispensables. L’acceptation vaccinale est plus fréquente chez les militaires, en raison de leur discipline, des contraintes institutionnelles et de leur respect des consignes hiérarchiques strictes. Leur comportement montre que l’adhésion à la vaccination peut être renforcée par des facteurs organisationnels et contextuels [5,6, 12].

L’interprétation des résultats de cette étude doit intégrer certaines limites, notamment un possible biais d’information lié à l’effet Hawthorne. Certains répondants ont pu orienter leurs réponses dans le sens du mieux-disant sur la Covid-19. Les enquêteurs ont tenté de corriger ce biais par des contre-vérifications en utilisant des questions indirectes ou reformulées pour tester la constance des réponses comme, par exemple, « vous dites avoir accepté de se faire vacciner contre la Covid 19 mais est-ce que vous pouvez nous montrer vos cartes de vaccination Covid-19 », puis « êtes-vous en mesure de persuader une autre personne de se faire vacciner? » ou « seriez-vous prêt à le faire? ». Les résultats restent exploitables et peuvent servir de base pour de futures recherches sur les causes d’acceptation de la vaccination. Toutefois, cette étude transversale analytique ne permet pas d’établir des liens de causalité directs. Elle offre néanmoins des données utiles sur l’acceptation et l’intention vaccinale, permettant d’élaborer des stratégies plus ciblées pour améliorer la couverture vaccinale, que ce soit pour la Covid-19 ou pour d’autres maladies épidémiques.

## Conclusion

L’étude menée dans la ZS Kokolo à Kinshasa visait à identifier les facteurs influençant l’acceptation de la vaccination contre la Covid-19. La majorité des habitants connaissait la maladie et la vaccination, principalement *via* la télévision. La quasi-totalité jugeait le vaccin efficace. Toutefois, ne pas avoir été confronté à des cas ou des décès liés à la Covid-19 expliquait une faible acceptation par certains. Par ailleurs, le contexte militaire de la ZS Kokolo a probablement permis d’augmenter significativement la couverture vaccinale, même si elle est restée inférieure aux attentes.

L’étude montre la nécessité de renforcer l’éducation sanitaire, d’améliorer l’accessibilité géographique et d’engager les leaders communautaires afin de transformer les intentions en actions concrètes. L’adhésion à la vaccination dépend d’une mobilisation multisectorielle et une communication continue adaptée aux réalités sociales de la communauté.

## Déclaration de consentement éclairé

Voir en Annexe.

## Comité éthique

N° d’approbation du comité éthique: ESP/ CE/129/2024

## Financement

Aucun financement externe. Enquête réalisée avec le financement local de la Zone de Santé Kokolo.

## Contributions des auteurs

MASAMBA BIKOKI Winnie: conception, analyses et rédaction.

NSINGA BUNGIENA Jean Claude: analyses et rédaction.

KAPE KALUME Jean Jacques: rédaction.

AMISI KENGEA Levis: auteur correspondant, conception, revue de la littérature, disponibilité des données, analyses, rédaction du rapport et de l’article.

## Déclaration de liens d’intérêt

Aucun lien d’intérêt n’a été déclaré.
