# Supplementary material for: Factors associated with acceptance of COVID-19 vaccination in the Kokolo health zone in Kinshasa, Democratic Republic of Congo (DRC)
Source: Med Trop Sante Int. 2025 Dec 22;5(4):mtsi.v5i4.2025.704. [Article in French] doi: 10.48327/mtsi.v5i4.2025.704 (PMC12948361; doi:10.48327/mtsi.v5i4.2025.704)
Supplement: Related texts

# Research article: Status of COVID-19 vaccination among staff at the Tengandogo University Hospital Center, Ouagadougou, Burkina Faso, June to December 2021

## Introduction

The Democratic Republic of the Congo (DRC) has faced significant challenges due to the ongoing spread of the novel coron avirus (SARS-CoV-2), particularly in Kinshasa and the Kokolo Health Zone (HZ) [1-8,10-13,16-20,22-24]. Since April 2021, the country has recorded 92,632 confirmed cases and 1,357 deaths, representing a case fatality rate of 1.47% [19]. Despite vaccination being introduced as the main strategy, uptake has remained low. Only 6.4% of Kinshasa residents have received the first dose, and 4.2% are fully vaccinated. All 27 health areas (HAs) in the Kokolo HZ have been affected, with a total of 3,829 cases and 28 deaths. The lack of vaccine uptake despite routine vaccination limited the effectiveness of the response. Unlike other countries, such as the Seychelles and Mauritius, the DRC did not achieve the desired coverage thresholds [4,8,12,21,24].

The goal of this study is to identify factors that promote acceptance of vaccination in order to tailor communication strategies and improve vaccination coverage. Specifically, the study sought to describe the sociodemographic and economic characteristics of heads of households or their spouses, determine their knowledge of, attitudes toward, and practices regarding, both the virus and the vaccine, and identify factors associated with vaccine acceptance.

## Methods

This cross-sectional analytical study was conducted in the Kokolo HZ from January 2021 to December 2024. This urban-rural and military zone is one of 35 health zones in the Kinshasa Provincial Health Division (DPS). The zone comprises 27 health areas (HAs) and covers a population of 653,645, with a central military hospital serving as the general referral hospital. The hospital had a treatment center for cases reported in the Funa district, one of the city of Kinshasa’s four districts. The Kokolo ZS did not have a 24/7 vaccination center for the administration of the SARS-CoV-2 vaccine due to a decision made by the national and provincial health authorities. However, there were 27 vaccination sites, one per HA, within the health center. These sites were open from 8 a.m. to 4 p.m. and were responsible for vaccinating approximately 50 people per day.

The study population consisted of household heads or their spouses, who constituted the statistical units.

Inclusion criteria included being 18 years of age or older and having lived in the household for more than six months.

Exclusion criteria included adults whose mental state prevented them from providing answers and those who did not consent to participate in the study. There were no exclusion criteria.

A minimum of 399 people were sampled using a three-stage probability sampling method. First, HAs were selected by simple random sampling using the list of HAs in the ZS as the sampling frame. In the second stage, community animation cells (CACs) were selected from the list of CACs in each selected HA. In each CAC, the third stage involved selecting households according to the sampling interval, and interviewing the head of the household or their spouse.

Data were collected through face-to-face interviews using a previously tested questionnaire. The Bandal ZS is adjacent to the Kokolo camp, with which it shares high population movement, including military personnel and civilians.

Data processing and analysis were carried out in accordance with confidentiality and anonymity, as well as quality control of data recorded in Excel 2016. The analysis was performed using Epi-Info 7.2.6.0 software.

Results were presented in tables with quantitative data shown as averages with standard deviations or medians with ranges and categorical data shown as proportions.

All necessary authorizations were obtained from the military and health authorities of the Kokolo HZ and its health facilities.

Participation in the survey was voluntary, and respondents were free to choose whether to participate in the study and answer the questions. Written informed consent was obtained from the respondents. The confidentiality of the collected information was guaranteed. Respondents could withdraw from the study at any time.

## Results

A total of 399 people were interviewed. More than half of them were women. Those over 54 years of age were in the minority. Nearly seven out of ten had a secondary education, and more than six out of ten were military personnel. There was no statistical difference between respondents with six or fewer children and those with more than six children in their households. More than six out of ten people walked to the vaccination site (Table I).

**Table I:** Sociodemographic characteristics of respondents

Variables	Headcount (n=399)	Ratio (%)
Gender		
female	201	50,4
male	198	49,6
Age (years)		
years	117	29,3
years	153	38,3
years	88	22,1
years	41	10,3
Marital status		
married	188	47,1
single	152	38,1
widowed	37	9,3
divorced	22	5,5
Level of education		
primary	214	53,6
secondary	132	33,1
higher	47	11,8
other (please specify)	6	1,5
Work before COVID-19		
military	261	65,4
military dependent	59	14,8
self-employed	49	12,3
unemployed	29	7,3
other (civilian personnel)	1	0,3
Current work		
military	263	65,9
military dependent	64	16,1
self-employed	45	11,3
unemployed	24	6,0
other (civilian personnel)	3	0,7
Number of children in the household		
≤ 6 children	201	50,4
> 6 children	198	49,6
Means of transportation		
walking	322	80,7
public transportation	62	15,5
personal transportation	4	1,0

Almost all respondents said they had heard of both the virus and the vaccine (Table II), with television being the main source of information. Seven out of ten respondents were aware of the importance and effectiveness of vaccination. A large proportion of respondents (90%) had knowledge about ways to combat the disease. Most were aware of the various side effects of vaccination (Table III). Nearly nine out of ten people had a good understanding of both the disease and the vaccine (Table IV).

**Table II:** Distribution of respondents according to their knowledge of COVID-19

Variables	Headcount (n=399)	Ratio (%)
Awareness of the existence of COVID-19		
yes	399	100
no	0	0
Source of information		
television	212	53,1
radio	94	23,6
social media	72	18,0
friends and family	54	13,5
community outreach	99	24,8
healthcare providers	31	7,8
Ways the virus spreads		
direct contact with someone who is sick	362	90,7
airborne transmission	308	77,2
contact with infected saliva droplets	227	56,9
contact with contaminated objects	64	16,0
Symptoms of COVID-19		
fever	381	95,5
cough	368	92,2
shortness of breath	367	91,9
headache	363	90,9
muscle pain	336	84,2
fatigue	312	78,2

**Table III:** Distribution of respondents according to their knowledge of COVID-19 vaccination

Variables	Headcount (n=399)	Ratio (%)
Awareness of the existence of COVID-19 vaccination		
yes	397	99,5
no	2	0,5
Source of information		
television	212	53,1
radio	99	24,8
social media	91	22,8
friends and family	82	20,5
community outreach	31	7,8
healthcare providers	54	13,5
Importance of vaccination		
aware	286	71,7
not aware	113	28,3
vaccine effectiveness		
strongly agree	197	49,4
agree	122	30,6
disagree	49	12,3
don’t know	31	7,8
measures to combat the virus		
face mask	374	93,7
regular hand washing	371	92,9
avoid handshakes	360	90,2
avoid physical contact	348	87,2
keep a distance of at least one meter	339	84,9
use your elbow	336	84,2
pray	317	79,4
use plants	258	64,7
go to a healthcare facility at the first sign of symptoms	254	63,7
be fully vaccinated	205	51,3
Side effects of vaccination		
headache	327	81,9
fever	324	81,2
aches/pains	305	76,4
diarrhea	264	66,2
nausea/vomiting	159	39,8
physical weakness	105	26,3

**Table IV:** Distribution of respondents according to their attitudes and practices regarding COVID-19

Variables	Headcount (n=399)	Ratio (%)
Believing in the existence of COVID-19	343	85,9
Reasons for disbelief		
never seen anyone die from COVID-19	58	14,5
never seen a case of COVID-19 in the HZ	51	12,8
never seen anyone recover from this disease	47	11,8
Predisposition of respondents to contract COVID-19 infection when exposed to the pathogen	266	66,7
Reasons for not being predisposed to contracting COVID-19 infection		
no contact with infected people	78	19,5
not being rich; it is a disease of the rich	63	15,8
COVID-19 does not exist	56	14,0
not living in affected HAs	39	9,8
Acceptance of the vaccine	319	79,9
Fully vaccinated people	184	46,1
Motivations for vaccination		
personal beliefs	83	20,8
by the community	37	9,3
by the health professionals	28	7,0
by the family members	19	4,8
by the military and civil authorities	17	4,3
Factors influencing vaccination		
community involvement	232	58,1
example set by authorities and community leaders	229	57,4
place of residence	229	57,4
rejection of fear	224	56,1
communication received from authorities and community leaders	223	55,9
socioeconomic status	221	55,4
level of education	219	54,9

More than eight out of ten people considered vaccination to be effective. The main reasons for not accepting vaccination were never having seen anyone die from or become infected with the virus. 25.1% of people were vaccinated, and the acceptance rate within the community was 9%. The most frequently cited factors influencing acceptance of the vaccine were not being afraid to be vaccinated with a newly marketed vaccine, the example set by authorities and community leaders, and the communication received from authorities and community leaders.

More than half of the respondents had seen someone get vaccinated against the virus, and over a third of those people were family members. Overall, nearly all respondents had never witnessed a death from the virus.

Of the various recommended measures to prevent the virus, regular handwashing was the most frequently cited. However, only 25% of respondents thought that vaccination would be an effective measure. Fear of complications was the main reason given for not accepting the vaccine. Nearly four in ten people cited raising awareness as a means of improving vaccine acceptance in the community (Table V).

**Table V:** Distribution of respondents according to their practices regarding vaccination

Variables	Headcount (n=399)	Ratio (%)
Measures taken to avoid contracting COVID-19		
wash your hands regularly	339	84,9
wear a mask	294	73,7
avoid shaking hands	272	68,2
keep a distance of at least 1 to 2 meters	261	65,4
avoid coughing	207	51,9
get vaccinated	184	46,1
avoid public places	167	41,8
reduce contact with unvaccinated people	135	33,8
difficulties encountered with vaccination		
fear of complications related to the vaccine	158	39,6
misinformation spread about vaccination	153	38,3
beliefs, customs, and traditions	137	34,3
discontinuous awareness among the population	132	33,1
good communication from apas* and community leaders	76	19,0
low socioeconomic status	52	13,0
long travel distance to reach healthcare facilities	44	11,0
insufficient vaccine stock	29	7,3
Proposals to improve acceptability		
targeted and sustained awareness-raising aimed at changing behaviors, strengthening knowledge, and mobilizing everyone around the response to COVID-19	159	39,9
manufacturing our own vaccines	84	21,1
development of inputs (medicines and protective equipment)	38	9,5
increase in the number of vaccination sites	19	4,8

* PPAs: Political and administrative authorities

Bivariate analysis revealed that six variables are related to vaccine acceptance: military status, knowledge about both the virus and vaccination, the involvement of political and administrative authorities (PAA), community leaders, awareness of the vaccine, and the proximity and accessibility of the vaccination site (Table VI).

**Table VI:** Relationship between factors and vaccine acceptance

Variables	Accepts the vaccine		Odds ratio	95% CI	p
	Yes (n=319)	No (n=80)			
Respondent gender					
female	160	41	1	(0,743-2,007)	0,431
male	159	39	1,221		
Age					
<54 years old	308	50	1	(0,046-2,823)	0,330
≥55 years old	II	30	0,359		
Occupation					
no occupation	18	6	1		
military dependent	58	6	4,500	(0,601-33,708)	0,143
military	207	56	18,50	(2,457-36,320)	0,005
self-employed	35	10	2,100	(0,817-34,615)	0,268
other (civilian personnel)	1	2	6,250	(1,016-38,448)	0,051
Knowledge of COVID-19					
aware	307	51	0,196	(0,099-0387)	< 0,001
unaware	12	29	1		
Knowledge of vaccination					
aware	302	61	4,036	(3,052-5,312)	0,003
unaware	17	19	1		
Involvement of PPAs, military authorities, and community leaders					
yes	262	51	1	(0,224-0,675)	< 0,001
no	57	29	0,393		
Awareness					
yes	299	62	4,036	(2,867-6,254)	< 0,001
no	20	18	1		
Proximity and accessibility of the site					
not remote	291	39	1	(0,265-0,731)	<0,001
remote	28	41	0,431		

* PPAs: Political and administrative authorities

The rating for military personnel was 18.5 times higher than for the unemployed. The rating was five times higher for people with knowledge about the virus than for those without. The rating was four times higher for people with knowledge about vaccination than for those without. A lack of involvement from PAA, military authorities, or community leaders reduced the likelihood of getting vaccinated by 2.6 times. Awareness increased the odds of getting vaccinated fourfold compared to those who were unaware.

Logistic regression revealed that three variables were significantly associated with vaccine acceptance: being in the military, having knowledge about the virus, and having knowledge about vaccination (Table VII).

**Table VII:** Distribution of respondents according to factors associated with vaccine acceptance

Characteristics	ORa (95%)	p (LR test)
Being military	3,09 (1,03-5,15)	0,005*
Knowledge about COVID-19	2,2 (1,33-3,26)	< 0,001*
Involvement of APAs, military authorities, and community leaders	9,11 (0,96-87,95)	0,056
Knowledge about vaccination	2,67 (1,05-4,65)	0,029*
Awareness	5,96 (0,88-11,01)	0,105
Proximity and accessibility of the site	7,04 (0,91-83,74)	0,057

Akaike information criterion value: 500. 954; AOR: adjusted odds ratio; LR: likelihood ratio; * significance

## Discussion

Most of the study population was aware of both the virus and the vaccine, with television being the main source of information (53%). Over 80% of respondents considered vaccination to be effective. However, the reluctance to be vaccinated was largely explained by never having seen a case of or death related to this virus in the ZS. Vaccine acceptance was three times higher among military personnel. It was also higher among those informed about the disease (2.2 times) and vaccination (2.6 times). Mbongopasi et al. found that 51% of people in Kinshasa considered television to be their main source of information [12]. However, the authors of this study suggest that there is a risk of misinformation due to the spread of a mixture of accurate and inaccurate information on the subject. A similar study in Uganda found that the main sources of information were international health organizations, such as the CDC and WHO [14-16].

Vaccination coverage in the Kokolo HZ reached 46%, primarily among individuals under 54 years old who lived near health centers where vaccination sites were located. While this rate exceeds the Congolese national average of 8.3%, it remains below expectations. Several factors explain this disparity in the ZS, including a lack of vaccination centers, limited mass campaigns, inadequate logistics, and frequent military movements. Reasons for vaccine refusal include the perceived absence of serious cases in the community and mistrust of vaccine efficacy. These reasons were also observed in the N’djili health zone, which is one of the municipalities of Kinshasa [2,3,7,17,19,24].

Skepticism fueled by rumors and a lack of confidence in the health system hinders the transition from intention to action. Clear messages about the safety and efficacy of vaccines are essential to remedy this. Military personnel are more likely to accept vaccines due to their discipline, institutional constraints, and strict adherence to hierarchical instructions. Their behavior demonstrates that adherence to vaccination can be reinforced by organizational and contextual factors [5,6,12].

When interpreting the results of this study, it is important to consider certain limitations, particularly the possibility of information bias related to the Hawthorne effect. Some respondents may have tailored their answers to create the best possible impression of their views on the pandemic. To correct for this potential bias, the investigators cross-checked responses using indirect or rephrased questions, such as, “You say you have agreed to be vaccinated against the virus, but can you show us your vaccination card?” and “Would you be willing to persuade someone else to get vaccinated? Or, “Would you be willing to do so?” Despite these limitations, the results remain usable and can serve as a basis for future research on the causes of vaccine acceptance. However, this cross-sectional analytical study does not allow for the establishment of direct causal links. Nevertheless, it provides useful data on vaccine acceptance and intention, enabling the development of more targeted strategies to improve vaccination coverage for both Covid-19 and other epidemic diseases.

## Conclusion

This study, conducted in the Kokolo HZ of Kinshasa, aimed to identify factors influencing acceptance of the Covid-19 vaccine. Most residents were aware of the disease and the vaccine, primarily through television. Almost all considered the vaccine to be effective. However, the low acceptance rate among some was explained by not having been confronted with cases or deaths related to the disease. Furthermore, the military context of the Kokolo HZ likely contributed to a significant increase in vaccination coverage, though it remained below expectations.

This study demonstrates the necessity of strengthening health education, improving geographical accessibility, and engaging community leaders to transform intentions into actions. Vaccination uptake depends on multisectoral mobilization and ongoing communication tailored to the community’s social realities.

## Informed consent statement

See Appendix.

## Ethics committee

Ethics Committee Approval Number: ESP/CE/129/2024

## Funding

No external funding. The survey was conducted with local funding from the Kokolo Health Zone.

## Authors’ contributions

MASAMBA BIKOKI Winnie: Design, analysis, and writing.

NSINGA BUNGIENA Jean Claude: analysis and writing.

KAPE KALUME Jean Jacques: Writing.

AMISI KENGEA Levis: Corresponding author; design; literature review; data availability; analysis; report; and article writing.

## Conflicts of interest

The authors declare no conflicts of interest related to this work

## Annexe: Questionnaire (outil de collecte des données)

Appendix: Questionnaire (data collection tool)
